# Cellular prion protein protects from inflammatory and neuropathic pain

**DOI:** 10.1186/1744-8069-7-59

**Published:** 2011-08-16

**Authors:** Vinicius M Gadotti, Gerald W Zamponi

**Affiliations:** 1 Department of Physiology and Pharmacology, Hotchkiss Brain Institute, University of Calgary, Calgary T2N 4N1, Canada; 2 Scientist of the Alberta Heritage Foundation for Medical Research and a Canada Research Chair in Molecular Neurobiology

**Keywords:** Prion protein, pain, knockout mice, NMDA receptor, spinal cord

## Abstract

Cellular prion protein (PrP^C^) inhibits *N*-Methyl-*D*-Aspartate (NMDA) receptors. Since NMDA receptors play an important role in the transmission of pain signals in the dorsal horn of spinal cord, we thus wanted to determine if PrP^C ^null mice show a reduced threshold for various pain behaviours.

We compared nociceptive thresholds between wild type and PrP^C ^null mice in models of inflammatory and neuropathic pain, in the presence and the absence of a NMDA receptor antagonist. 2-3 months old male PrP^C ^null mice exhibited an MK-801 sensitive decrease in the paw withdrawal threshold in response both mechanical and thermal stimuli. PrP^C ^null mice also exhibited significantly longer licking/biting time during both the first and second phases of formalin-induced inflammation of the paw, which was again prevented by treatment of the mice with MK-801, and responded more strongly to glutamate injection into the paw. Compared to wild type animals, PrP^C ^null mice also exhibited a significantly greater nociceptive response (licking/biting) after intrathecal injection of NMDA. Sciatic nerve ligation resulted in MK-801 sensitive neuropathic pain in wild-type mice, but did not further augment the basal increase in pain behaviour observed in the null mice, suggesting that mice lacking PrP^C ^may already be in a state of tonic central sensitization. Altogether, our data indicate that PrP^C ^exerts a critical role in modulating nociceptive transmission at the spinal cord level, and fit with the concept of NMDA receptor hyperfunction in the absence of PrP^C^.

## Background

The dorsal horn of spinal cord is an important site for pain transmission and modulation of incoming nociceptive information arriving from peripheral nociceptors [1,2]. Glutamate is the key neurotransmitter released by the primary afferent fibers [3,4] and plays an important role in nociceptor sensitization and in the modulation of allodynia [5]. Glutamate receptors (GluRs) such as *N*-methyl-*D*-aspartate (NMDA) receptors contribute in various ways to pain induction, transmission and control [5-7]. Consequently, NMDA receptor inhibitors exhibit antinociceptive and analgesic effects in rodents [8,9] as well in humans [10], however, their clinical use for the treatment of pain has been hampered by their CNS side-effects [11,12]. For this reason, strategies such as src interfering peptides have been proposed as ways to interfere with NMDAR hyperactivity in the pain pathway without affecting basal NMDA receptor function [13].

NMDA receptors are regulated by a plethora of cellular signaling pathways that could potentially be targeted for therapeutic intervetion [14]. Along these lines, our laboratory has recently shown [15] that NMDA receptor activity in mouse hippocampal neurons is regulated by cellular prion protein (PrP^c^). Specifically, NMDA receptors expressed in mice lacking PrP^C ^show slowed current decay kinetics, and spontaneous synaptic NMDA currents in pyramidal neurons displayed increased current amplitude [15]. We subsequently showed that PrP^C ^protects from depressive like behavior by tonically inhibiting NMDA receptor activity [16], thus suggesting that the altered NMDA currents in PrP^C ^null mice are associated with a clear behavioral phenotype. Given the important role of NMDA receptors in the afferent pain pathway, we hypothesized that absence of PrP^C ^may give rise to pain hypersensitivity. Here, we show that PrP^C ^null mice exhibit a decreased nociceptive threshold, both under basal conditions, as well as in models of inflammatory and neuropathic pain. These effects were reversed by treatment of the animals with the NMDA receptor antagonist MK-801, thus implicating NMDA receptor dysregulation in the observed pain phenotype.

## Results

### Mechanical and thermal withdrawal threshold of PrP^+/+ ^and PrP^-/- ^mice

**Figure 1:**
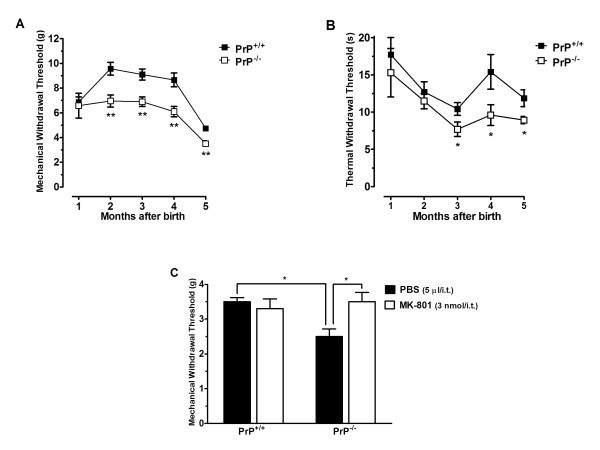
**Mechanical and thermal withdrawal threshold of PrP^+/+ ^and PrP^-/- ^mice**. Time course of basal mechanical (panel A) and thermal (panel B) nociceptive threshold of wild type or PrP^c ^null mice as a function of the age of the animal. (C) Effect of pretreatment of 3 months old mice with MK-801 (3 nmol/i.t.) on mechanical withdrawal threshold. Each point or column represents the mean ± S.E.M. (n = 5-6). *P < 0.05, **P < 0.01.

To determine if PrP^C ^plays a role in the transmission of pain signals, we compared nociception in wild type and PrP^C ^null mice. Paw withdrawal thresholds in response to mechanical and thermal stimuli were measured using the Dynamic Plantar Aesthesiometer (DPA) and Plantar Test devices, respectively. As shown in Figure 1, a blinded time-course analysis showed that PrP^C ^null mice exhibit significantly decreased mechanical and thermal withdrawal thresholds when compared to the wild-type group. Specifically, mechanical thresholds were significantly different in 2 month old animals (Figure 1A) whereas differences in thermal threshold appeared became statistically significant at an age of 3 months (Figure 1B). These differences were then maintained up to an age of 5 months, after which point the experiment was terminated. To ascertain whether this effect was mediated by spinal NMDA receptor hyperfunction, we intrathecally (i.t.) delivered 3 nmol of the NMDA receptor blocker MK-801 10 minutes prior to assessing mechanical withdrawal threshold. As shown in Figure 1C, MK-801 reversed the decreased mechanical withdrawal threshold of PrP^C ^null mice. Two-way ANOVA revealed a significant difference of genotype [F(2,57) = 5.6 P < 0.05] and genotype X-treatment interaction [F(1,43 = 5.5 P < 0.05)]. Altogether, these data indicate that the PrP^C ^inhibits nociceptive signalling through an NMDA receptor dependent mechanism.

### Nociceptive response of PrP^+/+ ^and PrP^-/- ^mice under acute stimulation

**Figure 2:**
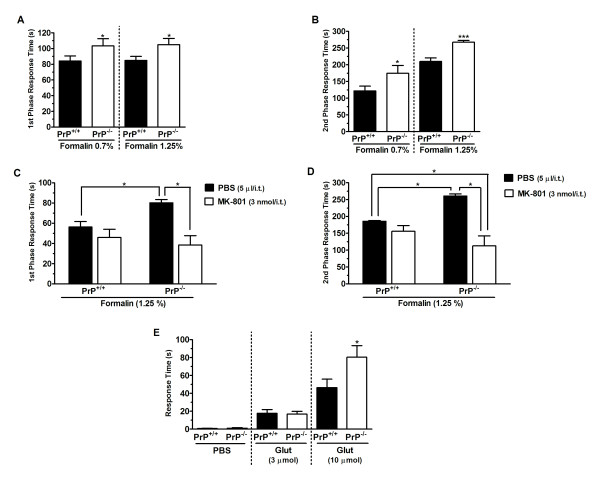
**Nociceptive response of PrP^+/+ ^and PrP^-/- ^mice under acute stimulation**. Nociceptive response of wild type or PrP^c ^null mice in the first (panel A) and second (panel B) phases of formalin-induced (0.7% or 1.25%) nociception. C, D Effect of pretreatment of animals with MK-801 (3 nmol/i.t.) for the first (panel C) and second (panel D) phases of the formalin response. E. Nociceptive responses of WT and null mice following intraplantar injection of glutamate. Each column represents the mean ± S.E.M. (n = 6-9). *P < 0.05, ***P < 0.001.

Next we used the formalin test to determine if PrP^C ^null mice display increased sensitivity to acute nociceptive stimulation. As shown in Figure 2, PrP^C ^null mice exhibited significantly elevated licking/biting time in both the first (Figure 2A) and second (Figure 2B) phases of nociception induced by formalin (0.7% or 1.25%). Treatment of mice with MK-801 (3 nmol/i.t., 10 min prior to testing) resulted in a significant reduction of the nociceptive behaviour of PrP^C-/- ^null mice for both the first (Figure 2C) and second (Figure 2D) phases of formalin-induced nociception. Two-way ANOVA revealed a significant difference of genotype [F(2,31) = 3.4, P < 0.05] and genotype X-treatment interaction [F(6,52) = 3.4, P < 0.05)] for the earlier (first) phase, and for the second phase (genotype [F(11,80) = 4.3, P < 0.05] and genotype X-treatment interaction [F(16,35) = 3.4, P < 0.001)]).

We also directly injected glutamate (3 μmol or 10 μmol) into the paws of wild type and null mice, and determined the time that the animals spent licking and biting over a 15 minute time course. As shown in Figure 2E, the higher dose of glutamate resulted in a significantly greater increase in response time compared to wild type animals.

### Nociceptive response of PrP^+/+ ^and PrP^-/- ^mice in response to NMDA treatment

**Figure 3:**
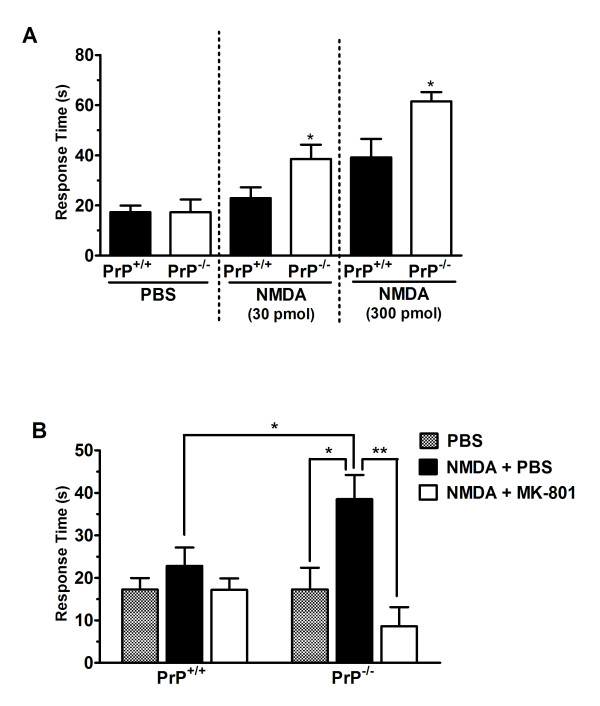
**Nociceptive response of PrP^+/+ ^and PrP^-/- ^mice in response to NMDA treatment**. (A) Nociceptive response of wild type or PrP^c ^null mice following intrathecal injection of NMDA (30 pmol/i.t. or 300 pmol/i.t.). Each column represents the mean + S.E.M. (n = 5-6). *P < 0.05. (B) Effect of MK-801 on the pain behaviour induced by intrathecal injection of NMDA (30 pmol). Each column represents the mean ± S.E.M. Control data (hatched bars) were obtained following i.t. injection of 5 μl PBS (i.e., the same route of delivery as for NMDA). NMDA data were obtained either following i.p. injection of 10 ml/kg PBS (black bars) or 0.005 mg/kg MK-801 (white bars). In this case, PBS serves as a control for MK-801. (n = 6-9). *P < 0.05, **P < 0.01.

To further investigate the involvement of spinal NMDA receptors in the decreased nociceptive threshold of PrP^C ^null mice we directly activated these receptors via i.t. NMDA injection. As shown in Figure 3A, PrP^C-/- ^mice exhibited a significantly higher nociceptive response (licking/biting) induced by different concentrations of intrathecally delivered NMDA. Interestingly, intrathecal injection of the lower dose of NMDA (30 pmol), which did not appear to affect wild-type mice, increased licking/biting time in PrP^C-/- ^null mice. These data suggest that hyperactivity of spinal NMDA receptors of PrP^C ^null mice may account for the decreased nociceptive sensitivity observed for those animals. As expected, treatment of animals with MK-801 (0.005 mg/kg, i.p., 30 minutes prior) prevented the effects of NMDA (Figure 3B).

### Nociceptive response of PrP^+/+ ^and PrP^-/- ^mice under neuropathic pain

**Figure 4:**
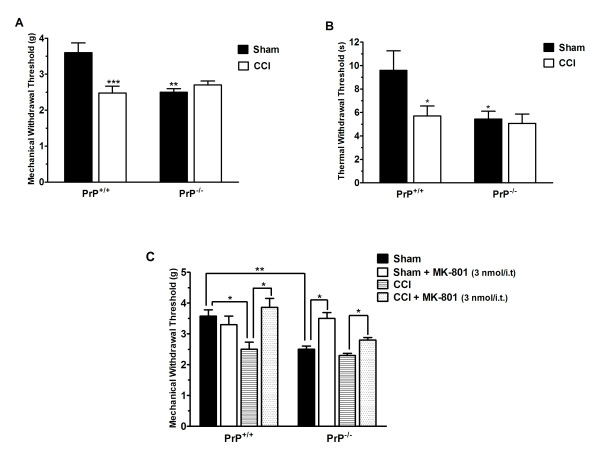
**Nociceptive response of PrP^+/+ ^and PrP^-/- ^mice under neuropathic pain conditions**. Mechanical (panel A) and thermal (panel B) withdrawal threshold of wild type or PrP^c ^null mice after CCI-induced neuropathy. The control data were obtained from sham-operated animals. (C) Effect of pretreatment of animals with MK-801 (3 nmol/i.t.) on mechanical withdrawal threshold. Each point or column represents the mean ± S.E.M. (n = 6-12). *P < 0.05, **P < 0.01, ***P < 0.001.

To determine if PrP^C ^modulates pain signalling under neuropathic conditions, we examined the response of wild type and null mice after sciatic nerve ligation (Chronic Constriction Injury-CCI). As shown in Figure 4, sciatic nerve ligation triggered decrease mechanical (Figure 4A) and thermal (Figure 4B) withdrawal thresholds in wild-type mice. Strikingly, this treatment did not further augment the already increased sensitivity of null mice to thermal and mechanical stimuli, as if PrP^C-/- ^mice behave as if they were tonically neuropathic. Moreover, treatment with MK-801 (3 nmol/i.t.) completely reversed mechanical allodynia induced by CCI in wild type mice and at least partially reversed the reduced mechanical threshold in nerve ligated PrP^C ^null mice (Figure 4C). A three-way analysis of variance revealed a statistical difference between genotype [F(3,4) = 9.13, P < 0.05], genotype X-treatment interaction [F(1,07) = 4.7, P < 0.01] and genotype × treatment × nerve injured interactions [F(1,75) = 11.4, P < 0.05].

## Discussion

It is well established that glutamate is involved in nociceptive processing in the spinal cord in both normal conditions and under certain pathological nociceptive processes [4,11,17] with NMDA receptors being the a key player in the binding of glutamate and activation of postsynaptic responses [18]. Under conditions of neuropathic pain, NMDA receptors are upregulated to mediate sensitization in second order neurons give rise to a phenomenon termed "wind up" [19-22] leading to allodynia and hyperalgesia [5]. Consequently, NMDA receptor antagonists have been known to decrease neuronal hyperexitability and reduce pain, and the efficacy of several NMDA receptor antagonists has been investigated in preclinical [8,9,23] and clinical pain studies [10]. Conversely, processes that augment NMDA receptor function would be expected to be pronociceptive.

We have recently shown that PrP^C ^physically interacts with NMDA receptors to inhibit NMDA receptor activity in the brain [15]. The absence of PrP^C ^resulted in increased amplitudes and durations of synaptic NMDA currents [15] whereas AMPA receptors were unaffected [24]. The data presented here are consistent with a similar NMDA receptor hyperfunction in the afferent pain pathway. Mice lacking PrP^c ^displayed increased nociceptive responses in thermal and mechanical tests, and showed an increased susceptibility to the development of inflammatory pain. Of note, an intrathecal dose of NMDA (30 pmol/i.t.), which does not induce in wild-type animals any pain behaviour *per se*, produced nociceptive behaviour in PrP^C ^null mice that was comparable to that exhibited by wild-type animals injected with a 10 fold higher dose of NMDA (300 pmol/i.t.). These data fit with NMDA receptor hyperactivity, and suggest that PrP^C ^null mice behave as if they are in a basal neuropathic pain state.

It is well established that nociceptive behaviour observed during first phase of formalin pain is a result of the direct chemical activation of peripheral nociceptors and by the release of local glutamate by the primary afferent, whereas the second phase results from central sensitization of dorsal horn neurons induced by primary afferent activity and peripheral inflammatory response [25]. However, the nociception caused by intraplnatar (i.pl.) injection of glutamate involves peripheral, spinal and supraspinal sites of action and is greatly mediated by both NMDA and non-NMDA receptors as well as by the release of nitric oxide [26]. Furthermore, it is also mediated by capsaicin-sensitive fibres and by release of neurokinins from sensory neurons that activate NK2 and B1 receptors [27]. NMDA receptor antagonists are effective in attenuating both phases of the nociception induced by formalin in rodents when delivered systemically [28,29] or spinally [30,31]. Moreover, NMDA receptor inhibitors reduced windup in dorsal horn neurons in animals with peripheral nerve injury [32-34], and NMDA receptor inhibition may impair wind-up in spinal neurons that relay C fiber input to the primary somatosensory cortex [35]. Our data showing sensitivity of the all of the observed PrP^C ^null mouse pain phenotypes to NMDA receptor blockers thus fit with these previous studies. Also in agreement with our data are results from Meotti and co-workers [36] showing that PrP^C ^null mice exhibited higher number of abdominal constrictions following intraperitoneal acetic acid injection when compared to wild-type mice. On the other hand, the same study showed that PrP^C ^null mice appeared to exhibit less pain in the tail-flick test, and had no difference in latency response in the hot-plate test, in contrast with our findings.

Although much attention has been focused on the infectious (misfolded) scrapie form of PrP^C ^(termed PrP^Sc^) it is becoming increasingly evident that normal PrP^C ^plays an important role in the normal physiology of the nervous system [37]. This includes neuroprotection [15,38] protection from epileptic seizures [39,40] and from development of depressive like behaviour [16] all of which have been linked to NMDA receptors. Our findings showing increased pain in PrP^C ^null mice fit with the concept of PrP^C ^playing a beneficial physiological role. It is widely recognized that misfolding of PrP^C ^into a β-sheet rich infectious prion mediates severe neurological phenotypes, such as Creutzfeldt-Jakob disease (CJD) and variant CJD [41-43]. In these disorders, normal PrP^C ^is progressively converted into infectious prions, which then form plaques and cause severe neurodegeneration, ultimately leading to death. It is possible that the conversion of normal PrP^C ^results in altered NMDA receptor currents in these patients, either by a reduction in the levels of normal PrP^C^, or by binding of misfolded PrP^Sc ^to the receptor complex. Such a mechanism may indeed fit with data showing that neuronal cultures infected with PrP^Sc ^are protected from cell death by NMDA receptor blockers [44]. In this context, it is interesting to note that a substantial fraction of new variant CJD patients show increased pain sensitivity [45] including limb pain [46]. Furthermore, there is a case report of a woman who developed vulvodynia despite normal vulvo-vaginal examination, and this patient was subsequently diagnosed with CJD [47]. While it is unknown as to whether this is due to augmentation of spinal NMDA receptor function in these patients due to compromised PrP^C ^regulation of the receptors, such a mechanism would be consistent with our observations in mice.

## Conclusion

In summary, our data indentify PrP^C ^as an important negative regulator of pain signalling. Considering that PrP^C ^physically interacts with the receptor complex to depress current amplitude, it may perhaps be possible to mimic this inhibition with small organic molecules interacting at the PrP^C ^interaction site on the receptor.

## Materials and methods

### Animals

All experiments were conducted following the protocol approved by the Institutional Animal Care and Use Committee (protocol #M09100) and all efforts were made to minimize animal suffering. Unless stated otherwise, 10 week old male mice (C57BL/6J wild type and PrP null weighing 25-30 g) were used. Animals were housed at a maximum of five per cage (30 × 20 × 15 cm) with food and water *ad libitum*. They were kept in 12 h light/dark cycles (lights on at 7:00 a.m.) at a temperature of 23 ± 1°C. All manipulations were carried out between 11.00 am and 3:00 pm. Different cohorts of mice were used for each test and each mouse was used only once. The observer was blind to the experimental conditions in the experiment examining the age dependence of the pain phenotype. Mice with a targeted disruption of the prion gene (PrP) of the Zürich 1 strain [48] were obtained from the European Mouse Mutant Archive (EM:0158; European Mouse Mutant Archive, Rome) and out-bred to generate PrP^-/- ^(PrP-null) littermates used in the experiments. Genotyping was performed by gel electrophoresis of PCR products obtained from genomic DNA that was isolated from tail samples. Primers and PCR parameters were similar to those used previously [48].

### Drugs and treatment

The following drugs were used in the study: *L*-glutamic acid hydrochloride, MK-801, *N*-methyl-*D*-aspartatic acid, Formaldehyde (Sigma Chemical Company, St. Louis, MO, USA). All drugs were dissolved in PBS. When drugs were delivered by the intraperitoneal (i.p.) route, a constant volume of 10 ml/kg body weight was injected. When drugs were administered by the intrathecal (i.t.) route, volumes of 5 μl were injected. Appropriate vehicle-treated groups were also assessed simultaneously. The choice of the doses of each drug was based on preliminary experiments in our laboratory.

### Formalin test

The formalin test is a widely used model that allows us to evaluate two different types of pain: neurogenic pain is caused by direct activation of nociceptive nerve terminals, while the inflammatory pain phase is mediated by a combination of peripheral input and spinal cord sensitization [49-51]. Animals received 20 μl of different concentrations of formalin solution (1.25% or 2.5%) made up in PBS injected intraplantarly (i.pl.) in the ventral surface of the right hindpaw. We observed animals individually from 0-5 min (neurogenic phase) and 15-30 min (inflammatory phase). Following i.pl. injection of formalin, the animals were immediately placed individually in observation chambers and the time spent licking or biting the injected paw was recorded with a chronometer and considered as nociceptive response. In experiemnts involving MK-801, mice were treated by intrathecal delivery 10 minutes prior to formalin injection (1.25%) at a dose of 3 nmol.

### Intraplantar glutamate injection

The procedure used was similar to that described previously [26]. Briefly, a volume of 20 μl of glutamate (3 nmol/paw or 10 nmol/paw prepared in PBS) was injected i.pl. into the ventral surface of the right hindpaw. Animals were observed individually for 15 min following glutamate injection. The amount of time spent licking the injected paw was recorded with a chronometer and was considered as nociceptive response.

### Intrathecal NMDA injection

To directly investigate the role of spinal glutamate receptors in the nociceptive behaviour observed for PrP^c ^knockout mice, we compared the nociceptive behavior of wild-type and PrP^c ^knockout mice after a single intrathecal injection of NMDA. Animals received an i.t. injection of 5 μl of NMDA solution. Injections were given to non-anaesthetized animals using the method described by Hylden and Wilcox [52]. Briefly, animals were restrained manually and a 30-gauge needle attached to a 25-μl microsyringe was inserted through the skin and between the vertebrae into the subdural space of the L5-L6 spinal segments. NMDA injections (30 pmol or 300 pmol) were given over a period of 5 seconds. The amount of time that animals spent biting or licking their hind paws, tail or abdomen was determined with a chronometer and considered as nociceptive response. In experiments involving MK-801, mice were treated by intraperitoneal delivery 30 minutes prior to NMDA injection at a dose of 30 pmol/site.

### Chronic constriction injury (CCI)-induced neuropathy

For neuropathic pain, we used a sciatic nerve injury model according to the method described by Bennett and Xie [53] with minor modifications. Briefly, mice were anaesthetized (isoflurane 5% indution, 2.5% maintenence) and the right sciatic nerve was exposed at the level of the thigh by blunt dissection through the biceps femoris. Proximal to the sciatic nerve trifurcation, about 12 mm of nerve was freed of adhering tissue and 3 loose ligatures (silk suture 6-0) were loosely tied around it with about 1 mm spacing so that the epineural circulation was preserved. In sham-operated rats, the nerve was exposed but not injuried. Mechanical and thermal withdrawal thresholds were determined 3 days after surgery. In another series of experiments, PrP^C ^null mice received MK-801 (3 pmol/i.t.) and mechanical withdrawal threshold was evaluated 10 min after drug delivery.

### Mechanical withdrawal threshold

To assess changes in sensation or in the development of mechanical allodynia, sensitivity to tactile stimulation was measured using the DPA (Ugo Basile, Varese, Italy). Animals were placed individually in a small enclosed testing arena (20 cm × 18.5 cm × 13 cm, length × width × height) with a wire mesh floor for 60 min. The DPA device was positioned beneath the animal, so that the filament was directly under the plantar surface of the foot to be tested. Each paw was tested three times per session. For experiment 1, the same cohort of both wild-type and PrP^C ^null mice were tested at age 1, 2, 3, 4 and 5 months. For neuropathic pain, testing was performed on the ispsilateral (ligated) paw before ligation (day 0) and then on the 3^rd ^day after ligation.

### Thermal withdrawal threshold

Thermal hyperalgesia was examined by measuring the latency to withdrawal of the hind paws from a focused beam of radiant heat applied to the plantar surface using a Plantar Test apparatus (Ugo Basile). Three trials each for the right hind paws were performed and for each reading, the apparatus was set at a cut-off time of 20 s. As with mechanical pain testing, the same cohorts of either wild-type or PrP^C ^null mice were used at 1, 2, 3, 4 and 5 months. Thermal withdrawal threshold was tested 1 day after they were used for mechanical testing. For neuropathic pain, testing was performed on the ispsilateral (ligated) paw before ligation (day 0) and then on the 4^th ^day after nerve injury.

### Statistical analysis

Data were presented as means ± SEM and evaluated by t-tests, two-way or three-way analysis of variance (ANOVA) followed by Tukey test when appropriate. A value of P < 0.05 was considered to be significant.

## Competing interests

The authors declare that they have no competing interests.

## Authors' contributions

VMG designed and performed experiments, data analysis and wrote the article. GWZ supervised the research project and edited the manuscript. The authors read and approved the final manuscript
